# Prevalence of symptoms of COVID-19 in the state of Rio Grande do Sul: results of a population-based study with 18,000 participants

**DOI:** 10.11606/s1518-8787.2021055004030

**Published:** 2021-11-12

**Authors:** Marilia Arndt Mesenburg, Pedro Curi Hallal, Ana Maria Baptista Menezes, Aluísio J D Barros, Bernardo Lessa Horta, Fernando Pires Hartwig, Nadege Jacques, Lucia Campos Pellanda, Alice de Medeiros Zelmanowicz, Daiane Oliveira Pereira Vergani, Edi Franciele Ries, Jenifer Harter, Jeovany Martínez-Mesa, Marcelo Carneiro, Sonara Lucia Estima, Thiago Gomes Heck, Mariangela Freitas da Silveira

**Affiliations:** I Universidade Federal de Pelotas Faculdade de Medicina Programa de Pós-Graduação em Epidemiologia Pelotas RS Brasil Universidade Federal de Pelotas. Faculdade de Medicina. Programa de Pós-Graduação em Epidemiologia. Pelotas, RS, Brasil; II Universidade Federal de Ciências da Saúde de Porto Alegre Departamento de Saúde Coletiva Porto Alegre RS Brasil Universidade Federal de Ciências da Saúde de Porto Alegre. Departamento de Saúde Coletiva. Porto Alegre, RS, Brasil; III Universidade de Caxias do Sul Caxias do Sul RS Brasil Universidade de Caxias do Sul. Curso de Enfermagem. Caxias do Sul, RS, Brasil; IV Universidade Federal de Santa Maria Centro de Ciências da Saúde Departamento de Saúde Coletiva Santa Maria RS Brasil Universidade Federal de Santa Maria. Centro de Ciências da Saúde. Departamento de Saúde Coletiva. Santa Maria, RS, Brasil; V Universidade Federal do Pampa Unidade Uruguaiana Uruguaiana RS Brasil Universidade Federal do Pampa. Unidade Uruguaiana. Curso de Enfermagem. Uruguaiana, RS, Brasil; VI Faculdade IMED Passo Fundo Escola de Medicina Programa de Pós-Graduação em Psicologia Passo Fundo RS Brasil Faculdade IMED Passo Fundo. Escola de Medicina. Programa de Pós-Graduação em Psicologia. Passo Fundo, RS, Brasil; VII Universidade de Santa Cruz do Sul Programa de Pós-Graduação Promoção da Saúde Santa Cruz RS Brasil Universidade de Santa Cruz do Sul. Curso de Medicina. Programa de Pós-Graduação Promoção da Saúde. Santa Cruz, RS, Brasil; VIII Universidade La Salle Canoas Canos RS Brasil Universidade La Salle Canoas. Curso de Enfermagem. Canos, RS, Brasil; IX Universidade Regional do Noroeste do Estado do Rio Grande do Sul Programa de Pós-Graduação em Atenção Integral à Saúde Ijuí RS Brasil Universidade Regional do Noroeste do Estado do Rio Grande do Sul. Programa de Pós-Graduação em Atenção Integral à Saúde. Ijuí, RS, Brasil; X Universidade Regional do Noroeste do Estado do Rio Grande do Sul Programa de Pós-Graduação em Modelagem Matemática e Computacional Ijuí RS Brasil Universidade Regional do Noroeste do Estado do Rio Grande do Sul. Programa de Pós-Graduação em Modelagem Matemática e Computacional. Ijuí, RS, Brasil

**Keywords:** COVID-19, Signs and Symptoms, Symptoms Hierarchy, COVID-19 Serological Testing, Predictive Value of Tests, Seroepidemiologic Studies

## Abstract

**OBJECTIVE:**

To evaluate the prevalence of reports of symptoms of COVID-19 among individuals with and without antibodies and identify those with greater capability to predict the presence of antibodies against SARS-CoV-2.

**METHODS:**

The study uses data collected in phases 5 to 8 of Epicovid-19-RS. The presence of antibodies against SARS-CoV-2 was evaluated by a rapid test. The occurrence of cough, fever, palpitations, sore throat, difficulty breathing, changes in taste and smell, vomiting, diarrhea, body pain, shaking, and headache since March 2020 was also evaluated. Then, the capability to predict the evaluated symptoms concerning the presence of antibodies was calculated.

**RESULTS:**

A total of 18,000 individuals were interviewed and 181 had antibodies against COVID-19 in phases 5 to 8. The proportion of asymptomatic individuals was 19.9% among participants with antibodies and 49.7% among those without antibodies. All symptoms were reported more frequently by individuals with antibodies. The division of the prevalence of symptoms among individuals with antibodies by the prevalence among individuals without antibodies showed the following prevalence ratios: for changes in smell or taste (9.1), fever (4.2), tremors (3.9), breathing difficulty (3.2) and cough (2.8 times). Anosmia and fever were the symptoms with a greater capability to predict the presence of antibodies.

**CONCLUSION:**

The prevalence of symptoms was higher among individuals with antibodies against SARS-CoV-2. The proportion of asymptomatic individuals was low. Altered smell or taste and fever were the symptoms that most predict the presence of antibodies. These results can help to identify probable cases, contributing to the clinical diagnosis and screening of patients for testing and isolation guidance in positive cases, especially in scenarios of the scarcity of diagnostic COVID-19 tests.

## INTRODUCTION

In December 2019, the first cases of COVID-19 were recorded and spread rapidly throughout the world, until, in March 2020, the World Health Organization characterized the disease as a pandemic^1,2^. The first studies on the symptomatology of patients with COVID-19 indicated that most cases were asymptomatic^3^ With the evolution of the pandemic and the identification of other symptoms related to the disease, studies conducted in different populations observed a great heterogeneity in the prevalence of symptomatic cases, ranging from 30 to 95%^3^. This variation depends on the symptoms investigated, the population studied, and the recall period, since many studies on symptomatology are conducted with patients in care for COVID-19, while others evaluated the general population. Probably the type of test used to identify the presence of antibodies also influences the results, because serological tests have less probability to detect less severe or asymptomatic cases, depending on the test and the time elapsed since infection^8^. Few population-based studies have evaluated the prevalence of COVID-19 symptoms. Studies evaluating the population distribution of symptoms of this disease are essential to understand its behavior, allowing the distribution of symptoms in the population to be known and not only in those who seek a health service, which tend to be the most severe cases.

Menezes et al. (2020) evaluated the prevalence of COVID-19 symptoms in the Brazilian population and showed that the most frequent symptoms were headache, changes in smell and/or taste, fever, cough, and myalgia. The proportion of participants with asymptomatic SARS-CoV-2 antibodies was 12.1%, among individuals without antibodies this proportion was 42.2%. Changes in smell and/or taste, fever, and myalgia were the symptoms with greater capability to indicate carriers and non-carriers of antibodies^4^. Other population-based studies have shown proportions of individuals with asymptomatic antibodies between 26% and 29%. The most frequent symptoms were changes in smell/taste, fever, tremors, and headache^9,10^.

In order to evaluate the prevalence of reported symptoms of COVID-19 among individuals with and without antibodies against SARS-CoV-2 and identify symptoms with greater capability to predict the presence of antibodies against this virus, this study analyzed data from a series of population-based surveys conducted in Rio Grande do Sul, Brazil.

## METHODS

The Epicovid-19-RS study is a series of serological surveys conducted in Rio Grande do Sul aiming to evaluate the magnitude and evolution of the COVID-19 epidemic. Nine cities were included in the study: Canoas, Caxias do Sul, Ijuí, Passo Fundo, Pelotas, Porto Alegre, Santa Cruz do Sul, Santa Maria e Uruguaiana. Except for Canoas, which was included in the sample as a representative of the metropolitan region of the capital, the other cities are the host cities of the intermediate sub-regions of Rio Grande do Sul, defined by the Brazilian Institute of Geography and Statistics (IBGE). In each stage, 4,500 individuals were evaluated.

Participants were selected by multi-stage sampling with probability proportional to size. In each city, 50 census tracts were drawn and, in each sector, 10 households were systematically selected. In each household, a resident was selected by simple random draw. If the selected resident was absent or refused to participate in the study, a second resident was drawn. In case of refusal of the second resident, the household was replaced by the one located immediately on the right side of the originally selected. Details of the study methodology were previously published^11^. Data collection was performed by trained interviewers, who used personal protective equipment (mask, glove, lab coat, and shoe covers), disposed of after each interview.

The occurrence of symptoms was evaluated with a structured questionnaire, as the recall of the presence of symptoms differed between steps one to four (15 days before this research) and five to eight (from March 2020), this study analyzed data from steps 5 to 8, performed on 06/26-28, 07/24-26, 08/14-16 and 09/04-06 of 2020. Cough, fever, palpitations, sore throat, difficulty breathing, changes in taste and smell, vomiting, diarrhea, body pain, tremors, and headache were the symptoms evaluated.

To measure the presence of antibodies against SARS-CoV-2, the WONDFO SARS-CoV-2 Antibody Test was used, which provides immediate results and employs the principle of lateral flow immunoassay for detection of antibodies against SARS-CoV-2. All tests with positive results were read by a second observer. The validation study conducted by the manufacturer showed a sensitivity of 86.4% and a specificity of 99.6%. Before the first stage of Epicovid-19-RS, the team of researchers validated the test in the study population, identifying the sensitivity of 77.1% and specificity of 98%^12^. With the emergence of evidence of a reduction in antibody titer over time, a second validation study was conducted, evidencing sensitivity of 63%^13^.

The statistical analysis included the description of the sample, the high estimation incidence of each symptom separately for individuals with a positive and negative rapid test, as well as the proportion of asymptomatic individuals. In order to identify which combinations of symptoms had the greatest capability to predict the result of the rapid test, conditional inference tree analysis was performed using binary recursive partitioning^14^. Statistical analyses were performed in the Stata16 program, considering the sample design effect and significance level of 0.05.

This study was approved by the National Committee of Ethics in Research (CONEP) (CAAE30721520.7.1001.5313). All participants signed the informed consent form.

## RESULTS

Data from 18,000 participants in the Epicovid-19-RS study were analyzed, out of which 181 tested positive for the presence of antibodies against SARS-CoV-2. The average age of the participants was 40 years (standard deviation 14.5). The majority were female (61%) and self-reported white skin color (76%). Table 1 shows the characteristics of the sample.

**Table 1:** Description of the sample of the Study Epicovid-19 - Rio Grande do Sul, steps 5 to 8.

Variable	n	%
Sex		
Female	10,955	60.9
Male	7,045	39.4
Age		
0–9	429	2.4
10–19	995	5.5
20–29	2,172	12.1
30–39	2,703	15
40–49	2,710	15.1
50–59	3,288	18.3
60–69	3,169	17.6
70–79	1,806	10
80 or above	728	4
Skin color/ethnicity		
White	13,382	76.1
Brown	2,704	15.4
Black	1,291	7.3
Yellow	144	0.82
Indigenous	88	0.5

The proportion of asymptomatic individuals was 49.7% (95%CI 48.6–50.9) among individuals without antibodies against SARS-CoV-2 and 19.9% (95%CI 14.9–26.1) among those with antibodies. Table 1 shows the prevalence of each symptom investigated according to the presence of antibodies. All symptoms were reported more frequently by individuals with antibodies against SARS-CoV-2, who reported 9.1 times more likely to present changes in smell or taste, 4.2 times more likely to present fever, 3.2 times more likely to present difficulty breathing, 3.9 times more likely to present tremors and 2.8 times more likely to present cough. All results were statistically significant (p-value < 0.05).

The Figure shows the results of the conditional inference tree analysis. Three of the 11 symptoms were selected: change in smell or taste, fever, and cough. Due to the low seroprevalence, in all terminal nodes, the prevalence was less than 15%. The 80% of the sample that did not report any of the three symptoms presented seroprevalence of 0.47%, whereas those who reported fever and changes in smell or taste had a seroprevalence of 13.4%.

**Figure:**
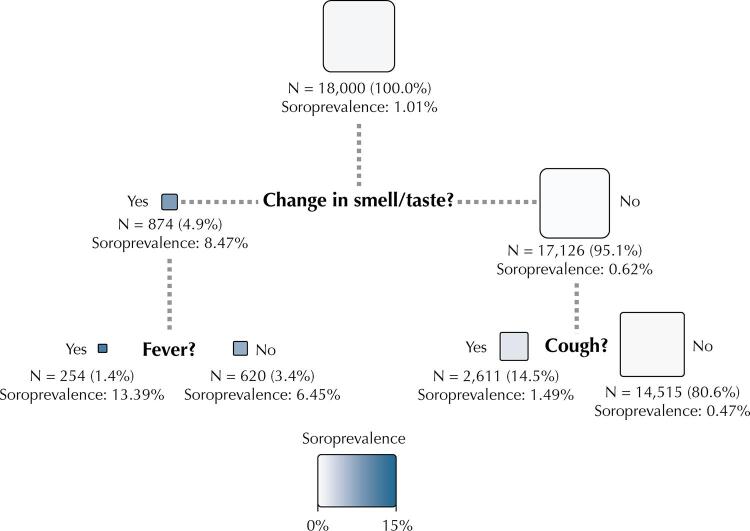
Conditional inference tree, study Epicovid-19 - Rio Grande do Sul, steps 5 to 8.

**Table 2:** Prevalence of COVID-19 symptoms among individuals with and without antibodies against SARS-CoV-2, in the study Epicovid-19 - Rio Grande do Sul, steps 5 to 8.

Symptom	Negative		Positive		PR	p
		
	n	Prevalence (95%CI)	n	Prevalence (95%CI)	(95%CI)	
Fever	1,278	7.2 (6.7–7.6)	54	29.8 (23.4–37.1)	4.2 (3.3–5.3)	< 0.001
Sore throat	3,282	18.4 (17.8–19.1)	65	35.9 (29.9–42.3)	1.9 (1.6–2.3)	< 0.001
Coughing	2,972	16.7 (15.9–17.4)	84	46.4 (39.9–52.9)	2.8 (2.4–3.2)	< 0.001
Breathing difficulty	943	5.3 (4.9–5.7)	31	17.1 (12.4–23.2)	3.2 (2.3–4.5)	< 0.001
Palpitations	1,113	6.2 (5.8–6.7)	24	13.3 (9.1–19.1)	2.1 (1.4–3.1)	< 0.001
Changes in smell/taste	800	4.5 (4.2–4.8)	74	40.9 (33.8–48.3)	9.1 (7.5–11.1)	< 0.001
Diarrhea	2,129	11.9 (11.4–12.5)	51	28.2 (22.1–35.2)	2.4 (1.9–2.9)	0.002
Vomiting	673	3.8 (3.5–4.1)	15	8.3 (5.1–13.2)	2.2 (1.4–3.5)	0.002
Pain in the body	2,060	11.6 (11.1–12.2)	61	33.9 (27.2–41.4)	2.9 (2.4–3.6)	< 0.001
Tremors	841	4.7 (4.4–5.1)	33	18.2 (12.8–25.3)	3.9 (2.8–4.5)	< 0.001
Headache	3,916	21.9 (21.1–22.9)	77	42.5 (36.2–49.1)	1.9 (1.7–2.2)	< 0.001

## DISCUSSION

The proportion of individuals with asymptomatic antibodies against SARS-CoV-2 was approximately 20%. All symptoms investigated were more frequent among participants with antibodies. The symptoms that presented the highest incidence ratio among carriers and non-carriers of antibodies were changes in smell and/or taste, fever, tremors, and breathing difficulty.

The results of this study are in line with the findings of a nationally representative study conducted with 33,205 Brazilians, whose results indicate that positive cases had 6.2 times more changes in smell/taste, 4.3 times more fever, as well as 3.3 times more reports of tremors^4^. Both in this study and the national study, the prevalence of asymptomatic patients was higher among negative cases. The prevalence of asymptomatic individuals without antibodies against SARS-CoV-2 was 20%, higher than the result of the national study (12.1%).

A population-based survey conducted in Maranhão found a prevalence of antibodies against SARS-CoV-2 of 40.4%, a result much higher than most studies with similar methodology. The same survey showed that the majority of antibody carriers reported some symptoms (62.2%), reporting more frequent changes in smell/taste and fever, corroborating the findings of this study^9^.

The Enecovid study, a national population-based serological survey conducted in Spain, found a seroprevalence of 5%. Among individuals with antibodies against SARS-CoV-2, the prevalence of asymptomatic individuals was 21.9% (19.1–24,9)^10^, which corroborates the findings of Epicovid-19-RS, going against the hypothesis raised at the beginning of the pandemic that most cases would be asymptomatic^5^. Considering the individuals with antibodies, among those who reported anosmia or at least three symptoms, the seroprevalence was 19.3% (17.7–21.0)^10^.

The probability of tests detecting the presence of antibodies against SARS-CoV-2 varies according to the time since infection^8,13,15,16^. Antibodies tend to be undetectable in the first days after contagion since their production usually occurs between 7 and 14 days after contamination. Thus, the test has limited diagnostic value of active infection, since it tends to detect only infections that occurred 15 days or more ago. If on the one hand very recent infections may not be detected, the detection of antibodies among individuals who have been infected longer also has limitations. The sensitivity of the test decreases significantly over time, especially among mild or asymptomatic cases, which introduces classification error, reducing differences in the prevalence of symptoms among carriers and non-carriers of antibodies^13^.

This study has other limitations, such as possible inaccuracy in the report of symptoms, since the presence of symptoms was asked since March 2020, and data collection occurred between June and September of the same year. Eventually, people who had COVID-19 longer may have reported fewer symptoms, which would decrease the proportion of symptomatic patients and the prevalence of each symptom. People who had more severe symptoms may have reported these manifestations more frequently compared to those who had mild symptoms, which would also lead to underestimation. On the other hand, given the characteristics of the test used^13^, it is necessary to consider the occurrence of false negatives, which would increase the proportion of individuals without antibodies with symptoms and decrease the difference in the prevalence of symptoms between infected and non-infected. The report of COVID-19-like symptoms produced by conditions other than SARS-CoV-2 infection may also have attenuated differences in the prevalence of symptoms among individuals with and without antibodies.

However, the strength of this study is to leverage the largest series of population-based serological surveys on COVID-19 conducted worldwide. The performance of serial surveys allowed the evaluation of different stages of the epidemic and related aspects in Rio Grande do Sul. Another positive point is that information on symptoms was collected in individuals with and without antibodies against SARS-CoV-2. Furthermore, the blinding of the interviewers and participants in relation to the test result, whose result was only disclosed after information collection, minimized the occurrence of detection bias.

The results of this study corroborate findings of other population-based studies that also showed a low proportion of asymptomatic individuals and changes in smell or taste as more specific symptoms for the disease and may help to identify probable cases, contributing to the clinical diagnosis and screening of patients for testing and guidance of isolation of positive cases, especially in scenarios of the scarcity of diagnostic tests of COVID-19. The contributions of this study to clinical practice are especially relevant in the current moment of the pandemic in Rio Grande do Sul and Brazil, which presents an explosion in the incidence and collapse of health services.
