# Supplementary material for: Prevalence of symptoms of COVID-19 in the state of Rio Grande do Sul: results of a population-based study with 18,000 participants
Source: Rev Saude Publica. 2021 Nov 12;55:82. doi: 10.11606/s1518-8787.2021055004030 (PMC8601401; doi:10.11606/s1518-8787.2021055004030)
Supplement: Translation

# Translation: Prevalência de sintomas característicos de covid-19 no Rio Grande do Sul: resultados de um estudo de base populacional com 18 mil participantes

**Authors:** Marilia Arndt Mesenburg, Pedro Curi Hallal, Ana Maria Baptista Menezes, Aluísio J D Barros, Bernardo Lessa Horta, Fernando Pires Hartwig, Nadege Jacques, Lucia Campos Pellanda, Alice de Medeiros Zelmanowicz, Daiane Oliveira Pereira Vergani, Edi Franciele Ries, Jenifer Harter, Jeovany Martínez-Mesa, Marcelo Carneiro, Sonara Lucia Estima, Thiago Gomes Heck, Mariangela Freitas da Silveira

**Affiliations:** I Brasil Universidade Federal de Pelotas. Faculdade de Medicina. Programa de Pós-Graduação em Epidemiologia. Pelotas, RS, Brasil; II Brasil Universidade Federal de Ciências da Saúde de Porto Alegre. Departamento de Saúde Coletiva. Porto Alegre, RS, Brasil; III Brasil Universidade de Caxias do Sul. Curso de Enfermagem. Caxias do Sul, RS, Brasil; IV Brasil Universidade Federal de Santa Maria. Centro de Ciências da Saúde. Departamento de Saúde Coletiva. Santa Maria, RS, Brasil; V Brasil Universidade Federal do Pampa. Unidade Uruguaiana. Curso de Enfermagem. Uruguaiana, RS, Brasil; VI Brasil Faculdade IMED Passo Fundo. Escola de Medicina. Programa de Pós-Graduação em Psicologia. Passo Fundo, RS, Brasil; VII Brasil Universidade de Santa Cruz do Sul. Curso de Medicina. Programa de Pós-Graduação Promoção da Saúde. Santa Cruz, RS, Brasil; VIII Brasil Universidade La Salle Canoas. Curso de Enfermagem. Canos, RS, Brasil; IX Brasil Universidade Regional do Noroeste do Estado do Rio Grande do Sul. Programa de Pós-Graduação em Atenção Integral à Saúde. Ijuí, RS, Brasil; X Brasil Universidade Regional do Noroeste do Estado do Rio Grande do Sul. Programa de Pós-Graduação em Modelagem Matemática e Computacional. Ijuí, RS, Brasil

**Keywords:** COVID-19, Sinais e Sintomas, Hierarquização dos Sintomas, Teste Sorológico para COVID-19, Valor Preditivo dos Testes, Estudos Soroepidemiológicos

## Abstract

**OBJETIVO:**

Avaliar prevalência de relato de sintomas característicos de covid-19 entre indivíduos com e sem anticorpos e identificar aqueles com maior capacidade de predição da presença de anticorpos contra o SARS-CoV-2.

**MÉTODOS:**

O presente estudo usa dados coletados nas fases de 5 a 8 do Epicovid-19-RS. A presença de anticorpos contra o SARS-CoV-2 foi avaliada por um teste rápido. Avaliou-se também a ocorrência dos sintomas tosse, febre, palpitações, dor de garganta, dificuldade para respirar, alterações no paladar e olfato, vômito, diarreia, dor no corpo, tremedeira e dor de cabeça, desde março de 2020. Então, calculou-se a capacidade de predição dos sintomas avaliados em relação a presença de anticorpos.

**RESULTADOS:**

Nas fases de 5 a 8, 18 mil indivíduos foram entrevistados e 181 apresentaram anticorpos contra covid-19. A proporção de indivíduos assintomáticos foi de 19,9% entre participantes com anticorpos e 49,7% entre aqueles sem anticorpos. Todos os sintomas foram relatados com maior frequência por indivíduos com presença de anticorpos. A divisão da prevalência de sintomas entre indivíduos com anticorpos pela prevalência entre indivíduos sem anticorpos evidenciou as seguintes razões de prevalência: para alterações de olfato ou paladar (9,1), febre (4,2), tremedeira (3,9), dificuldade respiratória (3,2) e tosse (2,8 vezes). Anosmia e febre foram os sintomas com maior capacidade de predizer a presença de anticorpos.

**CONCLUSÃO:**

A prevalência de sintomas foi maior entre indivíduos com anticorpos contra SARS-CoV-2. A proporção de indivíduos assintomáticos foi baixa. Alteração de olfato ou paladar e febre foram os sintomas que mais predizem a presença de anticorpos. Esses resultados podem auxiliar a identificação de casos prováveis, contribuindo para o diagnóstico clínico e triagem de pacientes para testagem e orientação de isolamento em casos positivos, especialmente em cenários de escassez de testes diagnósticos de covid-19.

## INTRODUÇÃO

Em dezembro de 2019 foram registrados os primeiros casos de covid-19 e disseminaram-se rapidamente pelo mundo, até que, em março de 2020, a Organização Mundial da Saúde caracterizou a doença como uma pandemia^1,2^. Os primeiros estudos sobre sintomatologia de pacientes de covid-19 indicavam que a maioria dos casos era assintomática^3^ Com a evolução da pandemia e a identificação de outros sintomas relacionados à doença, estudos realizados em diferentes populações observaram uma grande heterogeneidade na prevalência de casos sintomáticos, variando de 30 a 95%^3^. Essa variação depende dos sintomas investigados, da população estudada e do período recordatório, uma vez que muitos estudos sobre sintomatologia são realizados com pacientes em atendimento para covid-19, enquanto outros avaliaram a população geral. Provavelmente o tipo de teste utilizado para identificar a presença de anticorpos também influencie os resultados, pois testes sorológicos têm menor probabilidade de detectar casos menos graves ou assintomáticos, a depender do teste e do tempo decorrido desde a infecção^8^. Poucos estudos de base populacional têm avaliado a prevalência de sintomas de covid-19. Estudos que avaliem a distribuição populacional dos sintomas dessa doença são fundamentais para a compreensão do seu comportamento, permitindo que se conheça a distribuição dos sintomas na população e não apenas naqueles que procuram um serviço de saúde, que tendem a ser os casos mais graves.

Menezes e colaboradores (2020) avaliaram a prevalência de sintomas de covid-19 na população brasileira e mostraram que os sintomas mais frequentes foram cefaleia, alterações de olfato e/ou paladar, febre, tosse e mialgia. A proporção de participantes com anticorpos contra SARS-CoV-2 assintomáticos foi de 12,1%, entre os indivíduos sem anticorpos essa proporção foi de 42,2%. Os sintomas com maior capacidade de discriminar portadores e não portadores de anticorpos foram alterações de olfato e/ou paladar, febre e mialgia^4^. Outros estudos de base populacional evidenciaram proporções de indivíduos portadores de anticorpos assintomáticos entre 26% e 29%. Os sintomas mais frequentes foram alterações de olfato/paladar, febre, tremores e cefaleia^9,10^.

Com o objetivo de avaliar prevalência de relato de sintomas característicos de covid-19 entre indivíduos com e sem anticorpos contra SARS-CoV-2 e identificar sintomas com maior capacidade de predição da presença de anticorpos contra esse vírus, este estudo analisou dados de uma série de inquéritos de base populacional conduzidos no Rio Grande do Sul, Brasil.

## MÉTODOS

O estudo Epicovid-19-RS é uma serie de inquéritos sorológicos conduzidos no Rio Grande do Sul com o objetivo de avaliar a magnitude e evolução da epidemia de covid-19. Foram incluídas no estudo nove cidades: Canoas, Caxias do Sul, Ijuí, Passo Fundo, Pelotas, Porto Alegre, Santa Cruz do Sul, Santa Maria e Uruguaiana. À exceção de Canoas, que foi incluída na amostra como representante da região metropolitana da capital, as demais cidades são sedes das sub-regiões intermediárias do Rio Grande do Sul, definidas pelo Instituto Brasileiro de Geografia e Estatística (IBGE). Em cada etapa foram avaliados 4.500 indivíduos.

Os participantes foram selecionados por meio de amostragem em múltiplos estágios com probabilidade proporcional ao tamanho. Em cada cidade foram sorteados 50 setores censitários e, em cada setor, foram selecionados sistematicamente 10 domicílios. Em cada domicílio era selecionado um morador por sorteio aleatório simples. Caso o morador sorteado estivesse ausente ou se recusasse participar do estudo, um segundo morador era sorteado. Em caso de recusa do segundo morador, o domicílio era substituído pelo localizado imediatamente ao lado direito do selecionado originalmente. Detalhes da metodologia do estudo foram publicados previamente^11^. A coleta de dados foi realizada por entrevistadores treinados, que utilizavam equipamentos de proteção individual (máscara, luva, jaleco e propés), descartados após cada entrevista.

A ocorrência de sintomas foi avaliada com um questionário estruturado, como o recordatório da presença de sintomas diferiu entre as etapas um a quatro (15 dias antes desta pesquisa) e cinco a oito (a partir de março de 2020), esse estudo analisou dados das etapas 5 a 8, realizadas em 26-28/06, 24-26/07, 14-16/08 e 04-06/09 de 2020. Os sintomas avaliados foram tosse, febre, palpitações, dor de garganta, dificuldade para respirar, alterações no paladar e olfato, vômito, diarreia, dor no corpo, tremedeira e dor de cabeça.

Para medir a presença de anticorpos contra SARS-CoV-2 foi utilizado o teste rápido WONDFO SARS-CoV-2 Antibody Test, que fornece resultado imediato e emprega o princípio do imunoensaio de fluxo lateral para detecção de anticorpos contra SARS-CoV-2. Todos os testes com resultados positivos eram lidos por um segundo observador. O estudo de validação conduzido pelo fabricante apresentou sensibilidade de 86,4% e especificidade de 99,6%. Antes da primeira etapa do Epicovid-19-RS, a equipe de pesquisadores realizou a validação do teste na população em estudo, identificando sensibilidade de 77,1% e especificidade de 98%^12^. Com o surgimento de evidências de redução na titularidade de anticorpos ao longo do tempo, um segundo estudo de validação foi conduzido, evidenciando sensibilidade de 63%^13^.

A análise estatística incluiu a descrição da amostra, o cálculo da prevalência de cada sintoma separadamente, para indivíduos com teste rápido positivo e negativo, bem como a proporção de indivíduos assintomáticos. Com o objetivo de identificar quais combinações de sintomas apresentavam maior capacidade de predizer o resultado do teste rápido, foi realizada análise de árvore de inferência condicional usando particionamento recursivo binário^14^. As análises estatísticas foram realizadas no programa Stata16, levando em conta o efeito de delineamento amostral e nível de significância de 0,05.

**Tabela 2:** Prevalência de sintomas de covid-19 entre indivíduos com e sem anticorpos contra SARS-CoV-2, no estudo Epicovid-19 – Rio Grande do Sul, etapas de 5 a 8.

Sintoma	Negativos		Positivos		RP	p
		
	n	Prevalência (IC95%)	n	Prevalência (IC95%)	(IC95%)	
Febre	1.278	7,2 (6,7–7,6)	54	29,8 (23,4–37,1)	4,2 (3,3–5,3)	< 0,001
Dor de garganta	3.282	18,4 (17,8–19,1)	65	35,9 (29,9–42,3)	1,9 (1,6–2,3)	< 0,001
Tosse	2.972	16,7 (15,9–17,4)	84	46,4 (39,9–52,9)	2,8 (2,4–3,2)	< 0,001
Dificuldade respiratória	943	5,3 (4,9–5,7)	31	17,1 (12,4–23,2)	3,2 (2,3–4,5)	< 0,001
Palpitações	1.113	6,2 (5,8–6,7)	24	13,3 (9,1–19,1)	2,1 (1,4–3,1)	< 0,001
Alterações de olfato/paladar	800	4,5 (4,2–4,8)	74	40,9 (33,8–48,3)	9,1 (7,5–11,1)	< 0,001
Diarreia	2.129	11,9 (11,4–12,5)	51	28,2 (22,1–35,2)	2,4 (1,9–2,9)	0,002
Vômitos	673	3,8 (3,5–4,1)	15	8,3 (5,1–13,2)	2,2 (1,4–3,5)	0,002
Dor no corpo	2.060	11,6 (11,1–12,2)	61	33,9 (27,2–41,4)	2,9 (2,4–3,6)	< 0,001
Tremedeiras	841	4,7 (4,4–5,1)	33	18,2 (12,8–25,3)	3,9 (2,8–4,5)	< 0,001
Dor de cabeça	3.916	21,9 (21,1–22,9)	77	42,5 (36,2–49,1)	1,9 (1,7–2,2)	< 0,001

Este estudo foi aprovado pela Comissão Nacional de Ética em Pesquisa (CONEP) (CAAE30721520.7.1001.5313). Todos os participantes assinaram o termo de consentimento livre e esclarecido.

## RESULTADOS

Foram analisados os dados de 18 mil participantes do estudo Epicovid-19-RS, dos quais 181 apresentaram resultado positivo para presença de anticorpos contra SARS-CoV-2. A idade média dos participantes foi de 40 anos (desvio padrão 14,5). A maioria era do sexo feminino (61%) e de cor da pele autorreferida branca (76%). As características da amostra estão apresentadas na Tabela 1.

**Tabela 1:** Descrição da amostra do estudo Epicovid-19 – Rio Grande do Sul, etapas de 5 a 8.

Variável	n	%
Sexo		
Feminino	10.955	60,9
Masculino	7.045	39,4
Idade		
0–9	429	2,4
10–19	995	5,5
20–29	2.172	12,1
30–39	2.703	15
40–49	2.710	15,1
50–59	3.288	18,3
60–69	3.169	17,6
70–79	1.806	10
80 ou mais	728	4
Cor da pele/etnia		
Branca	13.382	76,1
Parda	2.704	15,4
Preta	1.291	7,3
Amarela	144	0,82
Indígena	88	0,5

A proporção de indivíduos assintomáticos foi de 49,7% (IC95% 48,6–50,9) entre indivíduos sem anticorpos contra SARS-CoV-2 e de 19,9% (IC95% 14,9–26,1) entre aqueles com anticorpos. A prevalência de cada sintoma investigado segundo presença de anticorpos é apresentada na Tabela 1. Todos os sintomas foram relatados com maior frequência por indivíduos com presença de anticorpos contra SARS-CoV-2, que relataram 9,1 vezes mais probabilidade de apresentar alterações de olfato ou paladar, 4,2 vezes mais febre, 3,2 vezes mais dificuldade respiratória, 3,9 vezes mais tremedeira e 2,8 vezes mais tosse. Todos os resultados foram estatisticamente significativos (valor p < 0,05).

A Figura exibe os resultados da análise da árvore de inferência condicional. Três dos 11 sintomas foram selecionados: mudança no olfato ou paladar, febre e tosse. Devido à baixa soroprevalência, em todos os nós terminais a prevalência foi inferior a 15%. Os 80% da amostra que não relatou nenhum dos três sintomas apresentou soroprevalência de 0,47%, enquanto aqueles que relataram febre e alteração do olfato ou paladar tiveram soroprevalência de 13,4%.

**Figura:** Árvore de inferência condicional, estudo Epicovid-19 – Rio Grande do Sul, etapas de 5 a 8.

## DISCUSSÃO

A proporção de indivíduos com anticorpos contra SARS-CoV-2 assintomáticos foi aproximadamente 20%. Todos os sintomas investigados foram mais frequentes entre participantes com presença de anticorpos. Os sintomas que apresentaram maior razão de prevalência entre portadores e não portadores de anticorpos foram alterações de olfato e/ou paladar, febre, tremores e dificuldade respiratória.

Os resultados desse estudo vão ao encontro dos achados de estudo nacionalmente representativo, realizado com 33.205 brasileiros, cujos resultados indicam que os casos positivos apresentavam 6,2 vezes mais alterações de olfato/paladar, 4,3 vezes mais febre, bem como 3,3 vezes mais relatos de tremores^4^. Tanto neste estudo quanto no estudo nacional, a prevalência de assintomáticos foi maior entre os casos negativos. A prevalência de assintomáticos entre os indivíduos sem anticorpos contra SARS-CoV-2 foi de 20%, superior ao resultado do estudo nacional (12,1%).

Um inquérito de base populacional realizado no Maranhão encontrou uma prevalência de anticorpos contra o SARS-CoV-2 de 40,4%, resultado muito superior à maioria dos estudos de metodologia semelhante. O mesmo inquérito evidenciou que a maioria dos portadores de anticorpos relatou algum sintoma (62,2%), relatando com maior frequência alterações de olfato/paladar e febre, corroborando os achados do presente estudo^9^.

O estudo Enecovid, inquérito sorológico nacional de base populacional realizado na Espanha, encontrou soroprevalência de 5%. Entre indivíduos com presença de anticorpos contra SARS-CoV-2, a prevalência de assintomáticos foi de 21,9% (19,1–24,9)^10^, o que corrobora os achados do Epicovid-19-RS e contrária a hipótese levantada ao início da pandemia, de que a maioria dos casos seriam assintomáticos^5^. Considerando os indivíduos com presença de anticorpos, entre aqueles que relataram anosmia ou pelo menos três sintomas, a soroprevalência foi de 19,3% (17,7–21,0)^10^.

A probabilidade de testes detectarem presença de anticorpos contra SARS-CoV-2 varia de acordo com o tempo desde a infecção^8,13,15,16^. Anticorpos tendem a ser indetectáveis nos primeiros dias após o contágio, uma vez que sua produção costuma ocorrer entre 7 e 14 dias após contaminação. Dessa forma, o teste possui valor diagnóstico limitado da infecção ativa, já que tende a detectar apenas infecções ocorridas há 15 dias ou mais. Se por um lado infecções muito recentes podem não ser detectadas, a detecção de anticorpos entre indivíduos que foram infectados há mais tempo também apresenta limitações. A sensibilidade do teste diminui de forma importante ao longo do tempo, principalmente entre casos leves ou assintomáticos, o que introduz erro de classificação, diminuindo diferenças na prevalência de sintomas entre portadores e não portadores de anticorpos^13^.

Esse estudo apresenta outras limitações, como possível inexatidão no relato dos sintomas, uma vez que foi indagada a presença de sintomas desde março de 2020 e a coleta de dados ocorreu entre junho e setembro do mesmo ano. Eventualmente, as pessoas que tiveram covid-19 há mais tempo podem ter relatado menos sintomas, o que diminuiria a proporção de sintomáticos e a prevalência de cada sintoma. Pessoas que tiveram sintomas mais graves podem ter relatado essas manifestações com maior frequência, comparado àqueles que tiveram sintomas leves, o que também levaria a uma subestimativa. Por outro lado, dadas as características do teste utilizado^13^, é necessário considerar a ocorrência de falsos negativos, o que aumentaria a proporção de indivíduos sem anticorpos com sintomas e diluiria a diferença na prevalência de sintomas entre infectados e não infectados. O relato de sintomas semelhantes aos de covid-19 produzido por outras condições que não a infecção por SARS-CoV-2 também pode ter atenuado diferenças na prevalência de sintomas entre indivíduos com e sem anticorpos.

Contudo, este estudo tem como pontos fortes tratar-se da maior série de inquéritos sorológicos de base populacional sobre covid-19 conduzidos em nível mundial. A realização de inquéritos seriados permitiu avaliar diferentes estágios da epidemia e aspectos relacionados no Rio Grande do Sul. Outro ponto positivo é que a informação sobre sintomas foi coletada em indivíduos com e sem anticorpos contra SARS-CoV-2. Ainda, o cegamento dos entrevistadores e participantes em relação ao resultado do teste cujo resultado só era divulgado após coleta das informações, minimizou a ocorrência de viés de detecção.

Os resultados deste estudo corroboram achados de outras pesquisas de base populacional que também evidenciaram baixa proporção de indivíduos assintomáticos e alteração de olfato ou paladar como sintomas mais específicos para a doença e podem auxiliar a identificação de casos prováveis, contribuindo para o diagnóstico clínico e triagem de pacientes para fins de testagem e orientação de isolamento de casos positivos, especialmente em cenários de escassez de testes diagnósticos de covid-19. As contribuições deste estudo para a prática clínica são especialmente relevantes no atual momento da pandemia no Rio Grande do Sul e no Brasil, que apresenta explosão da incidência e colapso dos serviços de saúde.
